# Establishment of a Live-Imaging Analysis for Polarized Growth of Conchocelis in the Multicellular Red Alga *Neopyropia yezoensis*

**DOI:** 10.3389/fpls.2021.716011

**Published:** 2022-02-16

**Authors:** Yuji Hiwatashi, Mizuho Shimada, Koji Mikami, Nagisa Takada

**Affiliations:** ^1^Graduate School of Food, Agricultural and Environmental Sciences, Miyagi University, Sendai, Japan; ^2^School of Food Industrial Sciences, Miyagi University, Sendai, Japan

**Keywords:** live-imaging, tip growth, cell division, cytoskeleton, conchocelis, red alga, *Neopyropia yezoensis*

## Abstract

A wide range of tip-growing cells in plants display polarized cell growth, which is an essential cellular process for the form and function of individual cells. Understanding of the regulatory mechanisms underlying tip growth in terrestrial plants has improved. Cellular processes involved in tip growth have also been investigated in some algae species that form filamentous cells, but their regulatory mechanisms remain unclear. In the macro red alga *Neopyropia yezoensis*, for which genome information has recently been released, the conchocelis apical cell exhibits tip growth and forms a filamentous structure. Here, we report a live-imaging technique using high-resolution microscopy to analyze the tip growth and cell division of *N. yezoensis* conchocelis. This imaging analysis addressed tip growth dynamics and cell division in conchocelis apical cells. The directionality and tip growth expansion were disrupted by the application of cytoskeletal drugs, suggesting the involvement of microtubules (MTs) and actin filaments (AFs) in these processes. A growing apical cell mostly contained a single chloroplast that moved toward the expanding part of the apical cell. Drug application also inhibited chloroplast movement, implying that the movement may be dependent on the cytoskeleton. The study determined that live-imaging analysis is a versatile approach for exploring the dynamics of tip growth and cell division in *N. yezoensis* conchocelis, which provides insights into the regulatory mechanisms underlying cellular growth in multicellular red algae.

## Introduction

A wide variety of tip-growing cells in fungi, animals, and plants exhibit polarized cell growth, which is a cellular process essential for the formation and function of the individual cell. Vesicles containing new cell wall components and membranes are secreted continuously and specifically from the interior into the apical dome. In addition, coupled with turgor pressure-driven cell expansion, that leads to restricted growth in this limited zone. The understanding of the regulatory mechanisms underlying tip growth in land plants has been advanced through the use of three main cell types: moss protonemata, root hairs, and pollen tubes of seed plants. The relationship between the cytoskeleton, anisotropic deposition of the cell wall, and polar exocytosis has been a subject of much interest in model plants, such as the eudicot *Arabidopsis thaliana* and the moss *Physcomitrium patens* (Rounds and Bezanilla, 2013; Bascom et al., 2018). Indeed, movement of nuclei and chloroplasts, as well as cytoskeletal dynamics, has already been observed in tip-growing living cells using fluorescent microscopy techniques in these organisms. Coupled with genetic manipulation, this approach is therefore a powerful tool for studying intracellular organellar movement and cytoskeletal dynamics in tip-growing cells (Rounds and Bezanilla, 2013).

In addition to land plants, filamentous cells are formed by tip growth in various algal taxa. In the green alga *Chara globlaris*, the rhizoids and protonemata grow by means of tip growth (Braun and Wasteneys, 1998; Braun and Richter, 1999). Prostrate filaments are formed *via* tip growth in the brown alga *Ectocarpus* sp., which belongs to the Stramenopiles (Rabille et al., 2019), a branch distinct from the Viridiplantae including green algae, mosses, and seed plants. Therefore, tip growth is common in the plants that produce filamentous structures. Cellular processes involved in tip growth have also been investigated in these algae species that form filamentous cells. Multicellular red algae exhibit filamentous cells that are formed by tip growth, but formation of filamentous cells has largely uncharacterized so far, and their regulatory mechanisms underlying cellular growth is unclear.

*Neopyropia yezoensis* is a marine multicellular red alga that is traditionally used as a material for dried seaweed (Nori in Japanese). This alga, previously referred to as *Pyropia yezoensis*, is proposed to be reclassified into the new genus *Neopyropia* based on the molecular phylogenetic analysis (Yang et al., 2020). Since *N. yezoensis* is an important aquatic resource, its culture conditions are well-established in the laboratory infrastructure, and a variety of genetic mutants have been isolated (Yan and Aruga, 2000; Yan et al., 2000; Niwa, 2010; Ding et al., 2016). Recently, the nuclear genome of *N. yezoensis* was sequenced (Nakamura et al., 2013; Wang et al., 2020), and genomic information has also been provided, which enables the identification of genes of interest. Transient gene transfer systems have also been established (Mikami, 2014). Therefore, *N. yezoensis* has recently attracted attention as a model plant for red algae.

*Neopyropia yezoensis* exhibits a triphasic life cycle consisting of gametophytes, sporophytes and conchosporophytes (Mikami et al., 2019), although traditional concept of the *N. yezoensis* life cycle is a diphasic consisting of gametophyte and sporophyte, in which conchosporangium was thought to be a part of sporophyte because of generation of it on sporophyte (Blouin et al., 2011; Takahashi and Mikami, 2017). However, the independence of the conchosporangium as a life cycle generation is inferred from the gene-expression analysis: conchosporangia exhibited unique gene expression profiles different from those of gametophytes and sporophytes and, notably, the KNOX gene encoding the TALE-homeobox transcription factor that is known as a master regulator determining the identity of life cycle generations was predominantly expressed only in the conchosporangia. These results indicates that the conchosporangium is distinct from gametophyte and sporophyte, and thus designated conchosporophyte (Mikami et al., 2019). Both conchocelis and conchosporangia as diploid sporophytes and conchosporophytes, respectively, are multicellular filamentous tissues that are established through tip growth and successive divisions of their apical cells located at the tips of the filamentous structure (Takahashi and Mikami, 2016; Mikami et al., 2019). Because it is easier to culture conchocelis than it is to culture conchosporangia, conchocelis apical cells of *N. yezoensis* are ideal for studies of tip growth.

In this study, using a live cell imaging with a high-resolution microscopy, we demonstrated the dynamics of tip growth of conchocelis apical cells. Pharmacological analysis with cytoskeletal drugs suggests that microtubules (MTs) and actin filaments (AFs) may be essential for proper polarized cell expansion and chloroplast movement during tip growth. Moreover, our time-lapse observation of cytokinesis revealed that a chloroplast was separated into two with furrowing of the septum. The usefulness of the live-imaging analysis is addressed to explore the regulatory mechanisms underlying tip growth and cell division in *N. yezoensis*.

## Materials and Methods

### Plant Material and Culture Condition

For routine subculturing, conchocelis of *N. yezoensis* strain U-51 (Mikami et al., 2019) were cultured in sterile artificial seawater (SEALIFE, Nihonkaisui Co., Ltd., Tokyo, Japan) supplemented with a nutritional mixture solution (Noriseed, Daiichi Seimo Co., Ltd., Kumamoto, Japan) as a nitrogen source, vitamins and trace metal elements. Briefly, 29.35 g of SEALIFE was completely dissolved in 1 L of distilled water and adjusted to pH 8.3 with 1 M KOH. After autoclaving, 1 mL of sterile-filtered Noriseed was added in 1 L of sterile artificial seawater. Conchocelis cells were grown in 300 mL of the medium at 20°C under 37 μmol m^–2^ s^–1^ in a long day period (16 h light/8 h dark). The medium was continuously bubbled with filter-sterilized air. For vegetative propagation, the conchocelis cells were collected every week and fragmented with forceps. The fragmented cells were inoculated in fresh (300 mL) medium and incubated under the same condition as described above.

For microscopic observation, Noriseed was used at a concentration of 0.2% or 0.1% as the nutrient mixture solution.

### Microscopy

The overall morphology of the conchocelis was observed under a stereo microscopy (SteREO Discovery. V12, Zeiss Microscopy, Jena, Germany) equipped with a cooled digital camera (DP70, Olympus, Tokyo, Japan).

For the initial imaging of living cells, conchocelis were cultured in artificial seawater supplemented with the nutrient solution in a glass-based dish (AGC Techno Glass, Co., Ltd., Tokyo, Japan) or plastic-based dish (μ-Dish 35 mm high, ibidi GmbH, Gräfelfing, Germany) for approximately 2 weeks. For stable imaging, conchocelis were inoculated in 0.5 mL of the medium in a plastic-based dish. After incubation at 20°C under 37 μmol m^–2^ s^–1^ in a long day period for 7 days, 1.5 mL of the medium was added in the dish, followed by incubation for 7–14 days. The conchocelis attached to a coverslip bottom of the plastic-based dish were observed.

Image acquisition for time-lapse observation was performed with an Axio observer Z1 inverted microscope with a 40 × /0.85-naumerical aperture (NA) objective lens (NEO FLUARPlan, Zeiss Microscopy) or a 63 × /1.40-NA objective lens (Plan-Apochromat, Zeiss Microscopy) equipped with a spinning disc-confocal unit CSU-X1 (Yokogawa electric Corporation, Tokyo, Japan) and a CCD camera (Axiocam 503 mono, Zeiss Microscopy) or a CMOS camera (ORCA-Flash4, Hamamatsu photonics, Hamamatsu, Japan).

A 488-nm excitation laser was used for autofluorescence of chloroplasts and FM1-43 fluorescence with collection of emission spectrum from 500 to 545-nm. A 561-nm excitation laser was used for autofluorescence of chloroplasts by collecting the 580–640-nm emission spectrum. Chloroplasts contain phycoerythrin that is excited by both 488-nm and 561-nm laser. The fluorescence signal specific to FM1-43 was detected through a comparison between the fluorescence signals with an excitation of 488 nm and 561 nm.

Time-lapse observation of chloroplasts during tip growth and cytokinesis was performed at 3-min intervals with Z-scan (1-μm intervals, 11 planes), using a 561-nm excitation laser. All z-sections were used to perform the maximum Z-projection for analyzing the tip growth. Of the 11 z-sections, five sections were used to process the maximum Z-projection of images of cytokinesis. Time-lapse observation of chloroplasts in the presence of cytoskeletal drugs was performed at 3-min intervals with Z-scan (1-μm intervals, 11 planes), using a 561-nm excitation laser. Of the 11 z-sections, six sections were used for processing the maximum Z-projection of the images. For long-term imaging of tip-growing conchocelis cells, time-lapse observations were performed using a 561-nm excitation laser. Image acquisition was performed at 20-min intervals with a Z-scan (1-μm intervals, 11 planes). Four z-sections were used to perform the maximum Z-projection.

For time-lapse imaging of FM1-43 fluorescence, image acquisition was performed at 10-min intervals with a Z-scan (1-μm interval, 11 planes) or 15-min intervals with a Z-scan (2-μm interval, 5 planes). All sections or some selected sections were used to perform the maximum Z-projection. For three-dimensional (3D) images of chloroplasts, z-sections (0.29-μm intervals, 35 planes or 0.31-μm intervals, 31 planes) were collected. For 3D-detection of FM1-43-labeled membranes in the septum, image acquisition was performed at with a Z-scan (0.31-μm interval, 31 planes). All sections were used to perform the maximum Z-projection.

For viable staining with Evans blue, an inverted microscopy IX73 (Olympus) with 40 × /0.6-NA objective lens (LUCPLFLN) equipped with a digital camera DP22 (Olympus) was used.

Image processing was performed using Fiji (Schindelin et al., 2012) (http://fiji.sc/Fiji). Chloroplast volume and cell volume was measured by using 3D ImageJ Suite (Ollion et al., 2013). Chloroplast movement was measured by using Manual Tracking plug-in. Five protruding edges of one chloroplast in conchocelis apical cells were manually recorded every 1 min and the velocity of each edge was calculated with at least 4 sequential time points. Finally, the chloroplast velocity (mean ± S.D.) was calculated as the average of at least five chloroplasts.

### Membrane Staining

To observe the plasma membrane and intracellular membrane, conchocelis were stained with FM1-43 (Biotium, Inc., CA, United States). Conchocelis were cultured in artificial seawater supplemented with 0.2% (v/v) Noriseed for 14 days. After removal of the medium, conchocelis were stained with 2 mL of the medium containing 5 μM FM1-43 and observed in the presence of FM1-43. For tip growth and cytokinesis, image acquisition was performed after the addition of FM1-43. For measurement of the fluorescence intensity of FM1-43 in growing apical domes, image acquisition started from 30 min after the addition of FM1-43 to stain plasma membrane sufficiently.

### Cell Viability

Conchocelis were stained with artificial seawater supplemented with 0.2% (v/v) Noriseed containing 0.05% (w/v) Evans blue (Nacalai Tesque, Inc., Kyoto, Japan) for 10 min. After staining, the cells were washed with the medium five times.

### Cytoskeletal Drug Treatment

Oryzalin and butamifos (FUJIFILM Wako Pure Chemical Corporation, Osaka, Japan) and latrunculin B (Abcam, Cambridge, United Kingdom) were dissolved in DMSO. After dilution to an appropriate concentration of these drugs, conchocelis cells were incubated in each well of a 24-well plate. After adding 2 mL of the medium containing these drugs into wells, cells were incubated at 20°C for 2 weeks under 37 μmol m^–2^ s^–1^ for a long day period. For time-lapse observation, conchocelis were incubated at 20°C for 2 weeks under 37 μmol m^–2^ s^–1^ in a long day period in 2.5 mL of the medium using the plastic-based dish as described above. After the medium was removed from the dish, 3 mL of the medium supplemented with 1 μM oryzalin, 10 μM butamifos, or 25 μM latrunculin B was added to the dish, and conchocelis were further cultured for 4 days in the presence of the drugs. When DMSO was used as the solvent, the highest DMSO concentration in the medium was 0.1% (v/v).

## Results

### Optimization of 3D-Imaging of Living Conchocelis Using a Confocal Laser Scanning Microscopy

To establish a live imaging system for growing conchocelis cells, dishes and media suitable for imaging living conchocelis cells were determined. Initially, conchocelis cells were cultured in artificial seawater containing 0.2% Noriseed using a glass-based dish and a plastic-based dish for observation with a microscopy. The growth of conchocelis cells in the plastic-based dish was better than that in the glass-based dishes, indicating that the plastic-based dish was appropriate to microscopic imaging (Supplementary Figure 1). After 14 days of culture, several filaments of conchocelis attached to a coverslip bottom of the plastic-based dish, and the conchocelis apical cells at the tips of these filaments grew continuously. Next, we determined the medium in which to examine the continuous growth of conchocelis cells using live imaging. We routinely used the medium with 0.2% Noriseed for live imaging because conchocelis cells were occasionally discolored in the medium supplemented with 0.1% Noriseed, when the conchocelis cells were cultured for 2 weeks (Supplementary Figure 1). For image acquisition with confocal laser scanning microscopy (CLSM), it is important to make the apical cells of conchocelis grow along the plastic coverslip bottom of the dish. To stably attach the growing apical cells to the coverslip bottom, the conchocelis placed on the center of the coverslip bottom was filled with medium (0.5 mL) and incubated for 1 week. During this incubation, several apical cells of conchocelis were attached to the coverslip bottom. Then, 1.5 mL of the medium was added to the conchocelis to grow continuously. Approximately 1–2 weeks after the culture, time-lapse observation with high-resolution Z-scan using CLSM was performed. The preparation of the conchocelis for imaging is summarized in Supplementary Figure 2.

### Time-Lapse Observation of Tip-Growing Apical Cells

Chloroplasts and vesicles were spatiotemporally organized in the apical dome of tip-growing cells in land plants; chloroplasts and vesicles were visualized by detecting autofluorescence of chloroplasts and fluorescence of intracellular membranes stained with FM1-43, respectively.

**FIGURE 1:**
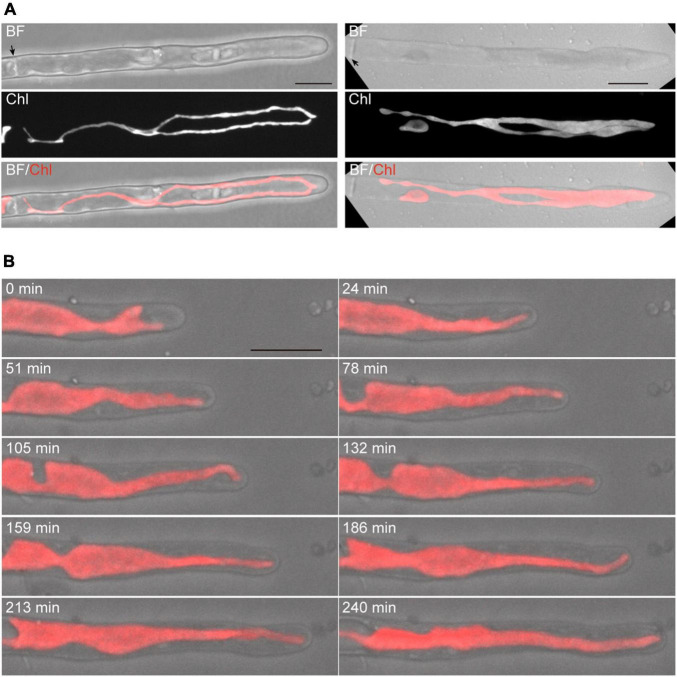
Dynamics of tip growth of conchocelis apical cells. **(A)** Images of conchocelis apical cells. The left and right panels show the images of an apical cell with one single chloroplast and two chloroplasts, respectively. The right and left images are maximum Z-projections of 35 planes (0.29-μm intervals) and 31 planes (0.31-μm intervals), respectively. The top, middle, and bottom panels show a bright field image, an autofluorescence image of chloroplasts, and a merged image of bright field (gray) and chloroplast autofluorescence (red), respectively. The Z-section images of chloroplast autofluorescence of the apical cell with one chloroplast (right panels) were subjected to the three-dimensional (3D) projection image (Supplementary Video 1). Arrows indicate the septum of conchocelis cells. **(B)** Serial observation of tip growth of a conchocelis apical cell. The images are maximum Z-projections of 11 planes (1-μm intervals). The images were acquired every 3 min (Supplementary Video 2). The bright field images (gray) and autofluorescence images (red) of a chloroplast are merged. Scale bars = 10 μm.

The Z-projection analysis with z-sections indicated that conchocelis apical cells have a large lobe-shaped chloroplast (Figure 1A and Supplementary Video 1). The apical cells had one or two chloroplasts but not cells with three or more chloroplasts (Figure 1A). Therefore, we counted the number of the apical cells including the number of each chloroplast. In conchocelis apical cells, 96.4% of cells (107 of 111 cells) had a single chloroplast and 3.6% of cells (4 of 111 cells) had two chloroplasts, indicating that most apical cells have a single large chloroplast that is aligned longitudinally throughout the cytoplasm of the cell. Using the FM1-43 membrane probe, plasma membrane and intracellular membrane components, such as vesicles, were clearly detected, while autofluorescence of chloroplasts was also detected by collecting the emission spectrum from 500 to 550-nm using a 488-nm excitation laser (Supplementary Figure 3).

**FIGURE 2:**
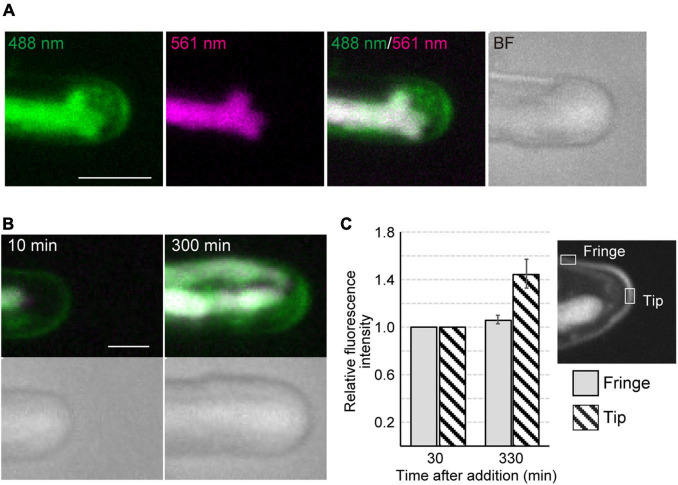
Dynamics of vesicles and plasma membrane during tip growth of conchocelis apical cells. **(A)** An apical dome stained with FM1-43. Vesicles and plasma membrane are visualized by detecting the FM1-43 fluorescence. The 488 nm and 561 nm panels indicate a fluorescence image (green) from both FM1-43 and a chloroplast acquired with a 488-nm emission laser and an autofluorescence image (magenta) from a chloroplast acquired with a 561-nm emission laser, respectively. The 488 nm/561 nm panel shows a merged image of fluorescence with a 488-nm emission laser (green) and with a 561-nm emission laser (magenta). In the 488 nm/561 nm panel, green color represents the FM1-43 fluorescence only, whereas magenta color (chloroplast autofluorescence) is overlaid with green color (FM1-43 fluorescence and chloroplast autofluorescence), resulting in white color. Note that magenta color is undetectable because all magenta is overlaid with green. The BF panel shows a bright field image. The images acquired about 10 h after addition of FM1-43 are shown. The images are maximum Z-projections of 11 planes (1-μm intervals). **(B)** Dynamics of vesicles and plasma membrane in the apical dome of a conchocelis apical cell stained with FM1-43. The left panel shows an image about 10 min after the addition of FM1-43. This image is set at 0 min. The images are maximum Z-projections of 11 planes (1-μm intervals). The top and bottom panels show the merged images of FM1-43 fluorescence (green) and chloroplast autofluorescence (magenta) and bright field images (gray), respectively. The images were acquired every 10 min. **(C)** Fluorescence intensity of FM1-43 signals in the apical dome. Measurements from a tip (gray bars) and fringe (hatched bars) of the apical dome (*n* = 9) are shown. The images acquisition started from 30 min after addition of FM1-43. The images were acquired every 15 min. Relative fluorescence intensity is relative to fluorescence intensity at 30 min. Error bars indicate SD. Scale bars = 5 μm.

Time-lapse microscopy observation showed that the expansion rate of the apical dome was measured at 0.065 μm/min (*n* = 48, SD = 0.025). During expansion of the apical dome, the protruding part of the chloroplast moved toward the apical expansion zone and was frequently distributed near plasma membrane on the tips of the apical dome (Figure 1B and Supplementary Video 2), indicating that chloroplasts predominantly expand toward the growing tips. Intracellular membrane components (e.g., vesicles) were abundant within the apical dome (Figure 2A). During the expansion, the fluorescence signal of the plasma membrane at the tip of the apical dome was more intense than that in the fringe of the apical dome (Figure 2B). The plasma membrane was uniformly labeled at 30 min after the addition of FM1-43, and the vesicles as well as the plasma membrane was labeled at 330 min. The fluorescence signals of FM1-43 in the plasma membrane of the apical dome were measured at both time points. The fluorescence intensity of FM1-43 in the tip at 330 min was approximately 1.4-fold higher than that at 30 min (Figure 2C). These results indicate that the membrane components predominantly accumulate in the tips of the growing apical dome.

### Disruption of Microtubules and Actin Filaments Leads to Aberrant Polarized Growth and Reduced Movement of Chloroplasts

**FIGURE 3:**
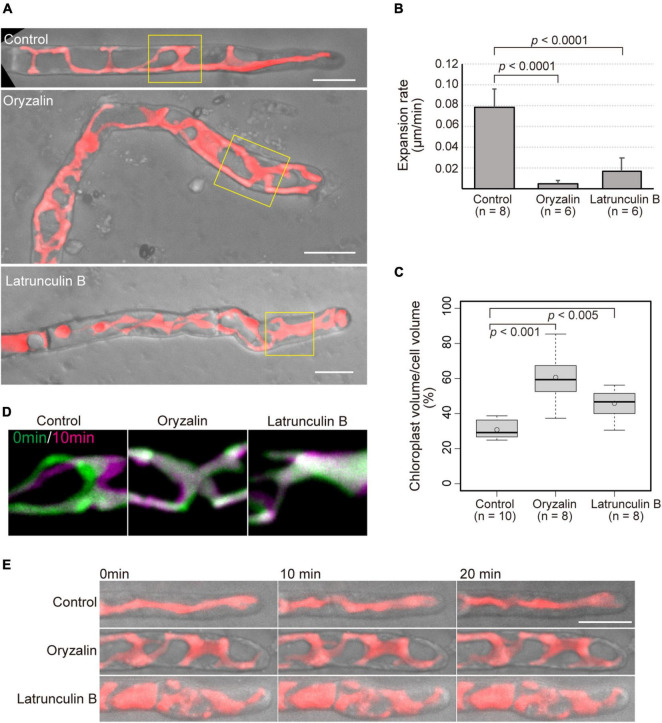
Application of cytoskeletal drugs leads to both misdirection and reduced expansion rate of tip growth, and reduced movement and expansion of chloroplasts. **(A)** Shape of conchocelis apical cells incubated with 1 μM oryzalin and 25 μM latrunculin B for 4 days. The images are maximum Z-projections of 6 planes (1-μm intervals). The bright field images (gray) and autofluorescence images (red) of chloroplasts are merged. Yellow boxes indicate regions for analyzing chloroplast movement in panel **(C)**. **(B)** Expansion rate of tip growth of conchocelis apical cells in the presence of 1 μM oryzalin and 25 μM latrunculin B. The expansion rate at the tips of the apical domes was measured by time-lapse imaging (interval 1 min, duration 20 min). *p* < 0.0001, unpaired *t*-test between control (DMSO) and cytoskeletal drugs. Error bars indicate SD. **(C)** Measurement of the ratio of chloroplast volume to cell volume of conchocelis cells incubated with 1 μM oryzalin and 25 μM latrunculin B. The graph shows box-plot medians and 25th/75th percentiles. Open circles indicate the mean ratio. *p* < 0.001 and 0.005, unpaired *t*-test between control (DMSO) and cytoskeletal drugs. **(D)** Movement of chloroplasts in the middle parts of conchocelis apical cells. The images are maximum Z-projections of 6 planes (1-μm intervals). The images were acquired every 1 min. The top panels show merged images of acquisition time at 0 min (green) and at 10 min (magenta). **(E)** Chloroplast movement of in the apical parts of conchocelis cells. The images are maximum Z-projections of 8 planes (1-μm intervals). The images were acquired every 1 min (Supplementary Video 3). The bright field images (gray) and autofluorescence images (red) of a chloroplast are merged. The top, middle, and bottom panels show merged images of cells incubated with 0.1% DMSO (Control), 1 μM oryzalin (Oryzalin), and 25 μM latrunculin B (Latrunculin B). Scale bars = 10 μm.

It is apparent that the cytoskeleton is essential for proper tip growth in land plants. To clarify the roles of MTs and AFs in the tip growth of conchocelis apical cells, conchocelis cells were incubated in the presence of a MT-depolymerizing drug, oryzalin, a MT-polymerization inhibitor, butamifos, and an actin polymerization inhibitor, latrunculin B. The conchocelis did not exhibited morphological alternations at the low concentration of these drugs, whereas their morphology was changed in the presence of oryzalin (1 μM) and butamifos (10 μM) for MT disruption, and latrunculin B (25 μM) for AF disruption (Supplementary Figure 4). The concentration of latrunculin B applied to conchocelis was higher than that to land plant cells, but conchocelis cells were viable at the concentration (Supplementary Figure 5). The dose-response analysis with latrunculin B suggests that conchocelis cells are less sensitive to latrunculin B than well-known angiosperms, such as *A. thaliana*. Indeed, the monospores, produced from the thallus of *N. yezoensis*, exhibited disorganization of AF and did not developed properly in the presence of 25 μM latrunculin B (Li et al., 2008). Relative high concentration of latrunculin B primarily causes AF disruption in *N. yezoensis* cells. While the control cells grew relatively straight, the cells treated with either oryzalin or latrunculin B produced wavy or curved apical cells (Figure 3A). The proportion of apical cells with wavy or curved shape in the presence of the drugs [67.6% (*n* = 37) and 54.8% (*n* = 31) in oryzalin and butamifos, respectively, and 62.5% (*n* = 72) in latrunculin B] was higher than that in the control treatment [3.2% (*n* = 62)]. The size of the conchocelis cell clusters under the application of the drugs was smaller than that of the control cell clusters in a dose-dependent fashion (Supplementary Figures 4,6), suggesting that the application of the drugs also affects the expansion rate of growth. An application of butamifos, also leaded to aberrant shape and reduced growth of conchocelis apical cells (Supplementary Figures 4A, 7). Thus, we measured the expansion rate of apical cell tips. The cells in the presence of oryzalin and latrunculin B showed 6% and 21% reductions, respectively, in the expansion rate compared to that of the control cells (Figure 3B). These results indicate that the application of oryzalin or latrunculin B leads to a loss of the control over the directionality of growth and a reduced expansion rate.

In the presence of the cytoskeletal drugs, the shape of the chloroplasts was also changed. In the growing apical cells in the control condition, the chloroplasts showed a lobed, streamlined shape, but in the presence of oryzalin or latrunculin B, the chloroplasts displayed a wavy mesh shape (Figure 3A). Chloroplast and cell volume were measured with 3D-reconstructed images. The ratio of chloroplast volume to cell volume under the application of the drugs was higher than that under the control condition (Figure 3C). This indicates that chloroplast expand over the cell under the presence of the drugs. Moreover, lowered movement of the chloroplasts was observed in the apical cells treated with the drugs, whereas the control cells exhibited that chloroplasts moved to the apical side (Figure 3D) and significantly moved (Figure 3E and Supplementary Video 3). The chloroplast velocity of the apical cells in the presence of the drugs [0.17 ± 0.05 μm/min (*n* = 5) and 0.24 ± 0.05 μm/min (*n* = 6) for oryzalin and butamifos, respectively, and 0.19 ± 0.03 μm/min (*n* = 8) for latrunculin B] was lower than that of the control cells [1.10 ± 0.23 μm/min (*n* = 13)]. The chloroplasts did not move much in the presence of drugs, indicating that the application of the drugs leads to reduced movement of chloroplasts in the cells.

### Dynamics of Cytokinesis and Chloroplast Division

**FIGURE 4:**
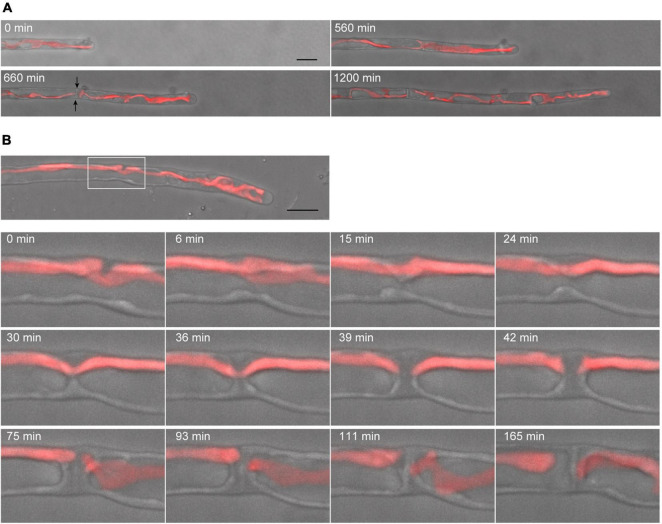
Dynamics of tip growth and cell division of conchocelis apical cells. **(A)** Cell division of a tip-growing apical cell in conchocelis. The images are maximum Z-projections of 4 planes (1-μm intervals). The images were acquired every 20 min. The bright field images (gray) and autofluorescence images (red) of chloroplasts are merged. Arrows indicate a newly formed septum between apical and subapical cells. **(B)** Cytokinesis of a conchocelis apical cell. The images are maximum Z-projections of 5 planes (1-μm intervals). The images were acquired every 3 min (Supplementary Video 4). The bright field images (gray) and autofluorescence images (red) of chloroplasts are merged. The top panel shows an image of a conchocelis apical cell. The lower panels show enlarged view of the furrow region (a white box in the top panel). Scale bars = 10 μm.

**FIGURE 5:**
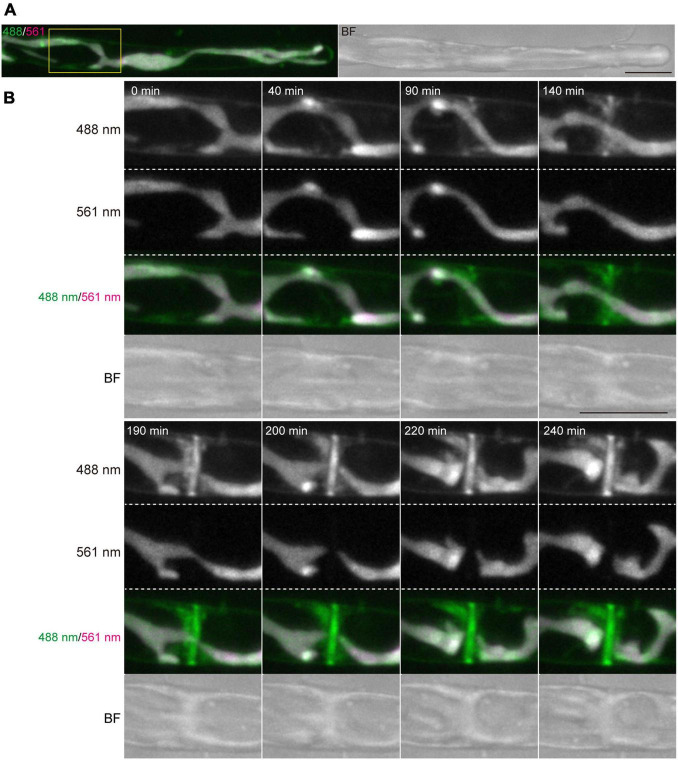
Dynamics of vesicles and plasma membrane during cytokinesis of conchocelis apical cells. **(A)** An apical cell stained with FM1-43. Vesicles and plasma membrane are visualized by detecting the FM1-43 fluorescence. The images are maximum Z-projections of 11 planes (1-μm intervals). The left and right panels indicate a merged image of fluorescence signals with 488-nm and 561-nm excitation mission laser (488/561) and a bright field image (BF), respectively. **(B)** Dynamics of membranes stained with FM1-43 during cytokinesis. The images of 11 planes (1-μm intervals). Dynamics of membrane and chloroplasts are shown in a white box in panel **(A)**. The image about 5 h after the addition of FM1-43 set at 0 min. The images were acquired every 10 min (Supplementary Video 5). The fluorescence image with 488-nm and 561-nm emission laser are referred as 488 nm and 561 nm, respectively. Scale bars = 10 μm.

**FIGURE 6:**
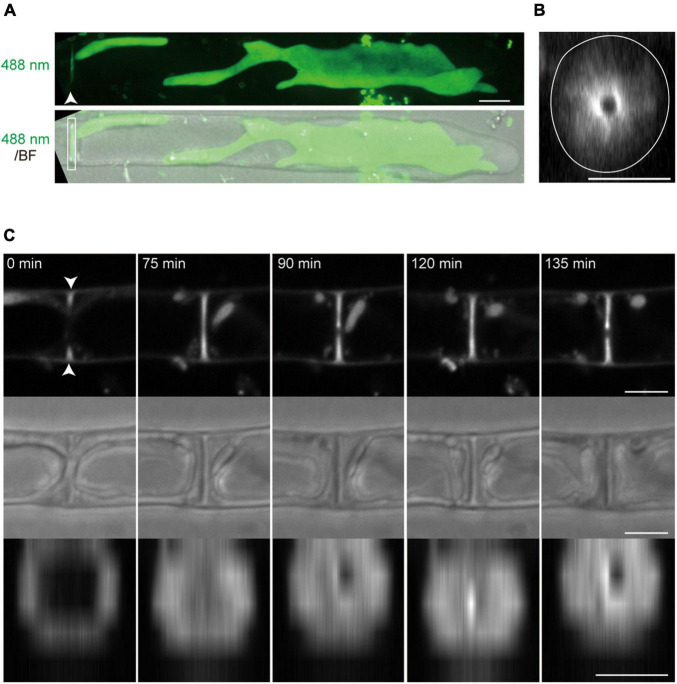
The membrane accumulation in the septum of conchocelis apical cells. **(A)** An apical cell stained with FM1-43. The images are maximum Z-projections of 35 planes (0.31-μm intervals). The top and bottom panels indicate an image of fluorescence signals with 488-nm excitation mission laser (488 nm) and a merged image of the fluorescence image and bright field image (BF), respectively. An arrowhead and box indicate the accumulation of the FM1-43-stained membranes and the septum, respectively. **(B)** A side-view of the image of fluorescence signals obtained by the 3D-reconstruction. This image was reconstructed with Z-slices at the position indicated by the arrowhead in panel **(A)**. A white circle line shows the cell margin. **(C)** The accumulation of membrane labeled with FM1-43 during cytokinesis. The top and middle panels indicate the images of fluorescence signals and bright images, respectively. These images are displayed after Z-projection of 3 plates (2-μm intervals) around the middle of the cell. The bottom panels indicate side-views of the images of fluorescence signals at the position shown by arrowheads in the top panel. The leftmost panel sets at 0 min. The images were acquired every 15 min. Scale bars = 5 μm.

Conchocelis tissue is a multicellular filament that is formed through tip growth and successive divisions of a conchocelis apical cell (Mikami et al., 2019). Thus, we performed time-lapse observations to investigate the dynamics of cell division. Long-term observation showed that a large chloroplast throughout the cytoplasm was divided into two at the position where cytokinesis occurred (Figure 4A). To obtain a more precise dynamics of cytokinesis and chloroplast division, image acquisition was performed at shorter time intervals. The chloroplast was separated by the furrowing septum, which was centripetally formed (Figure 4B and Supplementary Video 4). During cytokinesis of plant cells, vesicles are targeted to the division site and fused to build a new cell wall (Livanos and Muller, 2019). Thus, we investigated the accumulation of vesicles by detecting the FM1-43-staining membrane. The tubular vesicles accumulated in the future furrowing site and the vesicles were more abundant during furrow ingression (Figure 5 and Supplementary Video 5), indicating that the targeting of these vesicles occurs before the furrow ingression. We also found that the vesicles continuously accumulated in the septum after the cytoplasm was completely partitioned between the two daughter cells (Figure 5B). These results suggest that the targeting of the vesicles to the division site occurs prior to septum formation and continues after completion of the partition. After cytokinesis, The FM1-43-staining membranes accumulated in the septum (Figure 6A). The 3D-reconstruction image of the FM1-43-staining membranes at the septum indicated that the FM1-43-labeled membranes were excluded from the center of the septum, resulting in the formation of a single FM1-43 signal-free circular region (Figure 6B). Moreover, the FM1-43-labeled membrane predominantly accumulated around the circular region in a ring-like manner (Figure 6B). A single FM1-43 signal-free region was formed at the center of the septum during furrow ingression (Figure 6C), indicating the FM1-43-labeled membranes-free region was formed during cytokinesis.

## Discussion

In this study, we established a live-imaging technique using high-resolution microscopy to analyze the tip growth of conchocelis cells in *N. yezoensis*. Time-lapse imaging analysis addressed the dynamics of polarized expansion at the tips of the apical dome in conchocelis apical cells. The expansion rate of conchocelis apical cells is lower than that of tip-growing cells in land plants, such as pollen tubes and root hairs of the eudicot *A. thaliana* (Galway et al., 1997; Cheung et al., 2008) and caulonemal apical cells of the moss *Physcomitrium patens* (Hiwatashi et al., 2014), and tip-growing filamentous cells in the brown algae, such as *Ectocarpus* sp. (Rabille et al., 2019). In contrast, the expansion rate of conchocelis cells is higher than that of gymnosperm pollen tubes (Williams, 2008). Because the expansion rate of tip growth varies in the cell types and the taxa of plants, a comparison of the mechanism underlying conchocelis tip growth with that in other cells will help explain how molecular machinery responsible for managing the different expansion rates may evolve in a lineage-specific manner.

Most conchocelis apical cells have a large, lobed chloroplast that is distributed throughout the cytoplasm of the cells, indicating that the morphology and number of chloroplasts in *N. yezoensis* are different from those in land plants. In general, the chloroplasts of filamentous algae are present in lower numbers, and chloroplast morphology is variable in algae (Katalin, 2012). In *N. yezoensis*, each vegetative cell of the gametophytic thallus contains one chloroplast in the center (Ueki et al., 2009). Thus, the lower number and the variable shape of chloroplasts in *N. yezoensis* conchocelis cells indicate that these features are shared in both the sporophyte and gametophyte generation. In the expanding zone of the apical dome, the large chloroplast expanded toward the tips of the apical dome and were close to the apical dome. This distribution of the chloroplast is similar to that in the brown alga *Ectocarpus* because the chloroplast is distributed in the apical dome in *Ectocarpus* apical cells (Rabille et al., 2019). Moreover, the cells treated with the MT-depolymerizing drug, oryzalin, and an AF-polymerization drug, latrunculin B, showed that chloroplasts expanded over the cell and did not change their position inside the cells, suggesting that the shape and movement of chloroplasts are dependent on MTs and AFs. Therefore, it seems likely that the movement of chloroplasts toward the growing tips may be regulated by the cytoskeleton and its associated proteins. Indeed, light-induced chloroplast movement is regulated by the cytoskeleton and motor proteins, such as AF-based motor myosin and MT-based motor kinesin (Suetsugu et al., 2017). Interestingly, myosin genes were absent from the sequenced genome assembly of *N. yezoensis* and the bangiophyte red algae (Brawley et al., 2017; Goodson et al., 2021). One possible explanation is that kinesin motor proteins may be involved in the motility of chloroplasts during tip growth of conchocelis apical cells in an MT-dependent manner. However, the possibility that other cellular components participate in the chloroplast movement could not be excluded. Thus, it is a challenging task to clarify the regulators involved in the movement of chloroplasts in *N. yezoensis*.

Application of cytoskeletal drugs leads to reduced expansion of apical cells and aberrant direction of the conchocelis cells, suggesting that both MTs and AFs are indispensable for proper tip growth of conchocelis apical cells. MTs and AFs are fundamental components that play critical roles in tip growth in land plants (Rounds and Bezanilla, 2013). Our results indicate that these cytoskeletal components are essential for proper tip growth in red algae. Kinesins accumulate in tip-growing cells, mirroring many critical functions (e.g., MT organization and transport of cellular components on MTs) during tip growth. Considering the absence of myosin genes in the genome of *N. yezoensis*, kinesin may play a role in promoting the expansion and determining the direction of the growth as a motor protein. Imaging of intracellular membranes stained with FM1-43 revealed that vesicles accumulated in the growing apical dome, suggesting that the vesicles may be targeted at the tips of the apical dome. Vesicle transport may be dependent on kinesin-based activity. The investigation of MT organization and kinesins in the growing apical dome will offer insight into their precise roles in polarized tip growth.

Our imaging analysis of cell division in conchocelis apical cells revealed that a large chloroplast was divided through furrowing cytokinesis. This result is consistent with a previous study in another cell type such as spermatogonial cells (Ueki et al., 2009), indicating that chloroplasts are synchronously divided by the furrowing septum. The tubular vesicles predominantly accumulated in the cytokinetic site prior to chloroplast division, suggesting that new cell walls were synthesized by ingression of the septum. Thus, the chloroplasts could be physically cleaved by the furrowing septum. An alternative explanation is that a supramolecular machine with ring-shaped contractile complexes may function the division of chloroplasts. In land plants and green algae (i.e., Viridiplantae), chloroplast division is performed by constriction of a ring-like division complex called plastid-division (PD) rings (Miyagishima, 2011; Yoshida, 2018a). In the unicellular red alga *Cyanidioschyzon merolae*, plastid division and cell division can be highly synchronized (Yoshida, 2018b). The PD ring forms the main framework of the division machinery at the division site of the plastid in the unicellular red alga. It is still an open question whether the PD ring forms at the division site of the plastid in *N. yezoensis*.

During cytokinesis, a single circular region without the FM1-43-labeled intracellular membranes was formed in the center of the septum. In the septum of red algae, pit connections are formed between daughter cells during cytokinesis (Hawkins, 1972; Ueki et al., 2008). A pit connection is usually a single circular structure, suggesting that the circular region without the FM1-43-labeled membrane may be located at the pit connection. This 3D-imaging system can be available for analyzing formation of pit connections in living cells.

In conclusion, we have established a live-imaging system for studying tip growth and cell division in the multicellular red alga, which this system could be a useful experimental approach to improve our understanding of the molecular basis of tip growth and cell division in the near future.

## Data Availability Statement

The original contributions presented in the study are included in the article/Supplementary Material, further inquiries can be directed to the corresponding author/s.

## Author Contributions

YH conceived the experiments. YH, MS, and NT performed the experiments, collected, and analyzed the data. KM contributed to data analysis. YH and KM wrote the manuscript. All authors contributed to the article and approved the submitted version.

## Conflict of Interest

The authors declare that the research was conducted in the absence of any commercial or financial relationships that could be construed as a potential conflict of interest.

## Publisher’s Note

All claims expressed in this article are solely those of the authors and do not necessarily represent those of their affiliated organizations, or those of the publisher, the editors and the reviewers. Any product that may be evaluated in this article, or claim that may be made by its manufacturer, is not guaranteed or endorsed by the publisher.

## Supplementary Material

Supplementary Video 1 The three-dimension (3D) image of a chloroplast in a conchocelis apical cell corresponding to Figure 1.

Supplementary Video 2 Dynamics of tip growth of a conchocelis apical cell corresponding to Figure 1.

Supplementary Video 3 Chloroplast movement in the apical parts of conchocelis apical cells incubated with cytoskeletal drugs corresponding to Figure 3.

Supplementary Video 4 Dynamics of cytokinesis of a conchocelis apical cell corresponding to Figure 4.

Supplementary Video 5 Dynamics of cytokinesis of a conchocelis apical cell stained with FM1-43 corresponding to Figure 5.
