# Supplementary material for: Establishment of a Live-Imaging Analysis for Polarized Growth of Conchocelis in the Multicellular Red Alga Neopyropia yezoensis
Source: Front Plant Sci. 2022 Feb 16;12:716011. doi: 10.3389/fpls.2021.716011 (PMC8888420; doi:10.3389/fpls.2021.716011)
Supplement: Supplementary file 6 [file Data_Sheet_1.pdf]

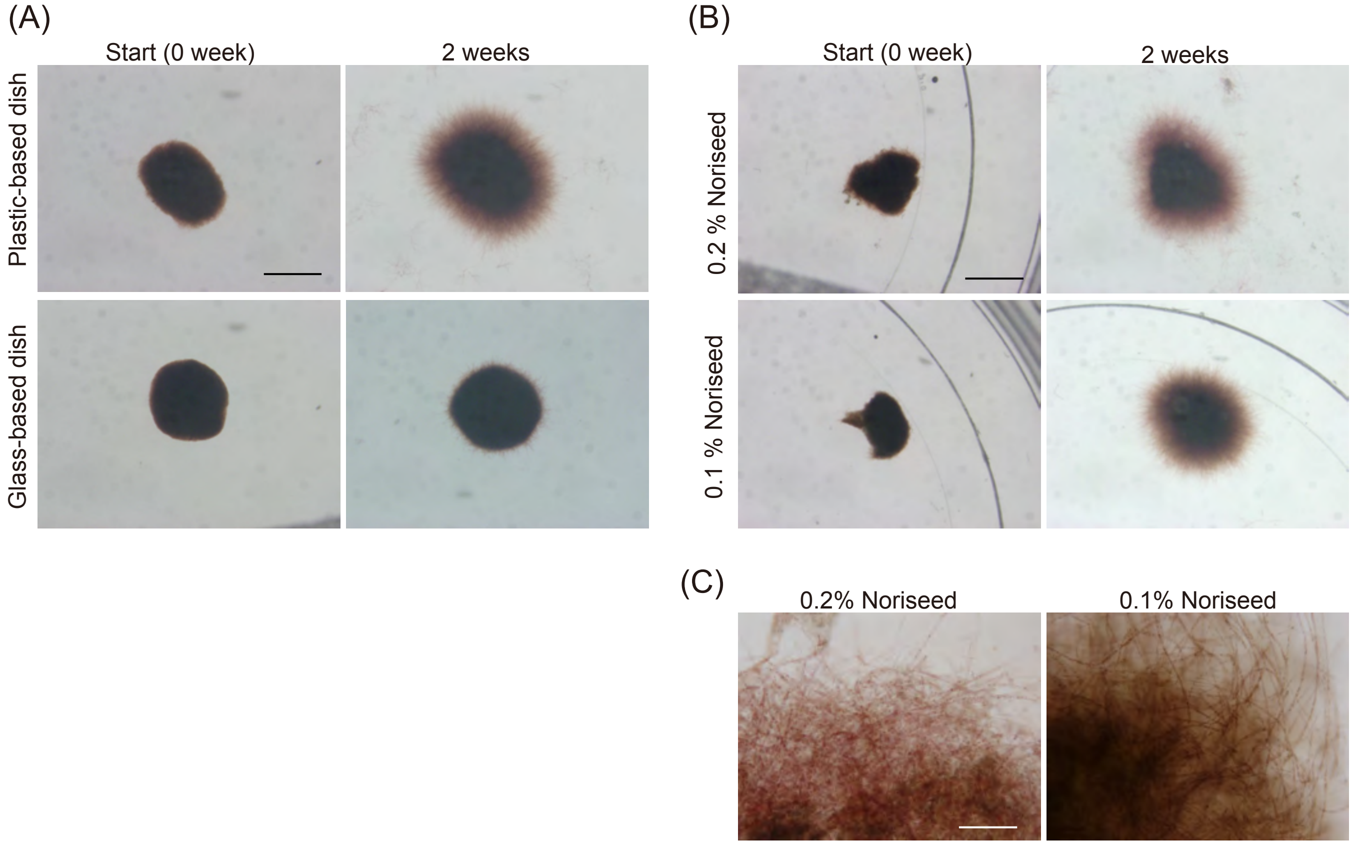

Supplementary Figure 1. The culture condition for imaging of conchocelis cells.
(A) Conchocelis cell clusters cultured for 2 weeks in plastic-based dishes (upper panels) and glass-based dishes (lower panels). (B) Conchocelis cell clusters cultured for 2 weeks in the medium supplemented with 0.2% Noriseed (upper panels) and 0.1% Noriseed (lower panels). Scale bars = 2 mm. (C) Conchocelis filaments cultured for 2 weeks in the medium supplemented with 0.2% (left panel) and 0.1% (right panel) of Noriseed. A scale bar = 0.2 mm.

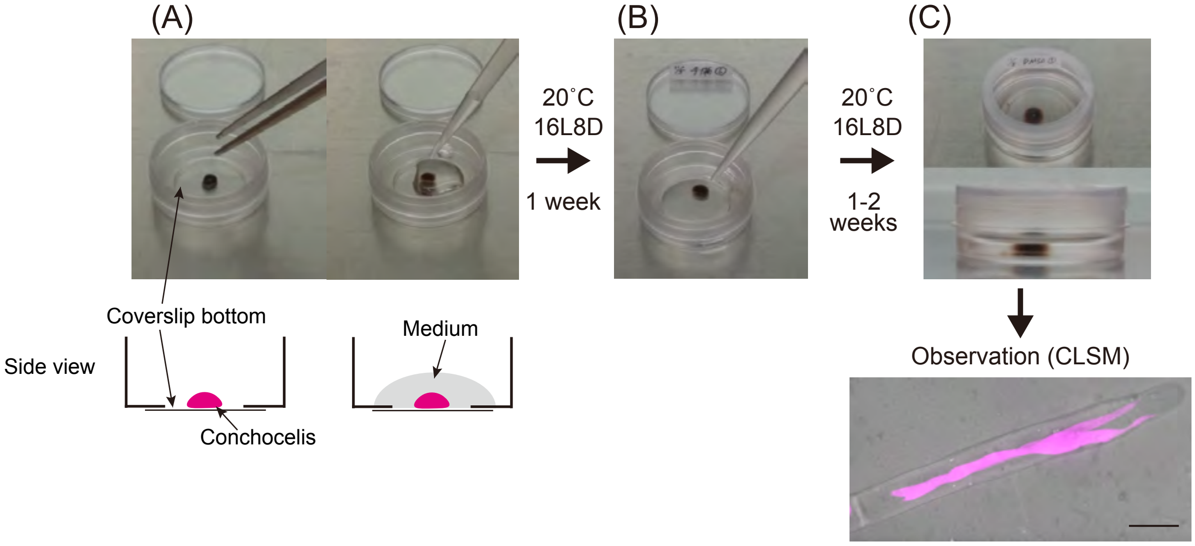

Supplementary Figure 2. Preparation of conchocelis cells for imaging.
(A) Conchocelis cells in a plastic-based dish. Cells were placed in the center of the coverslip of the dish with forceps, and 0.5 mL of medium was added into the dish. Schmatics indicates a side view of the plastic-based dish. (B) Growing conchocelis cells after 1 week of incubation. The dish was filled with an additional 1.5 ml of medium. (C) Conchocelis cells for imaging with a confocal laser scanning microscopy (CLSM). The cells were observed from 1 to 2 weeks after the addition of medium.

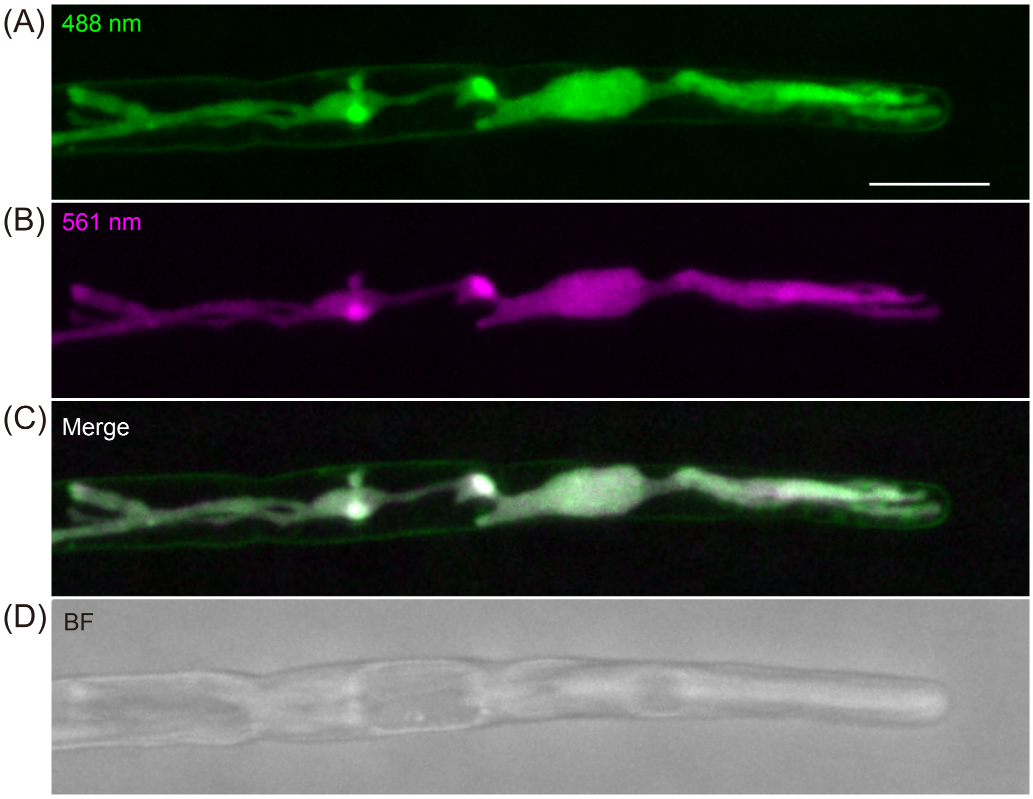

Supplementary Figure 3. Fluorescence images of plasma membrane and intracellular membranes stained with FM1-43 in a conchocelis apical cell.

(A) The fluorescence image detected by a 488-nm excitation laser. Both FM1-43 fluorescence and chloroplast autofluorescence were detectable. (B) The chloroplast autofluorescence image detected by a 561-nm excitation laser. (C) The merged image. The signals detected by a 488-nm excitation laser (green) are merged with the signals detected by a 561-nm excitation laser (magenta). The images are maximun Z-protection of 11 planes (1 µm-intervals). A scale bar = 10 µm.

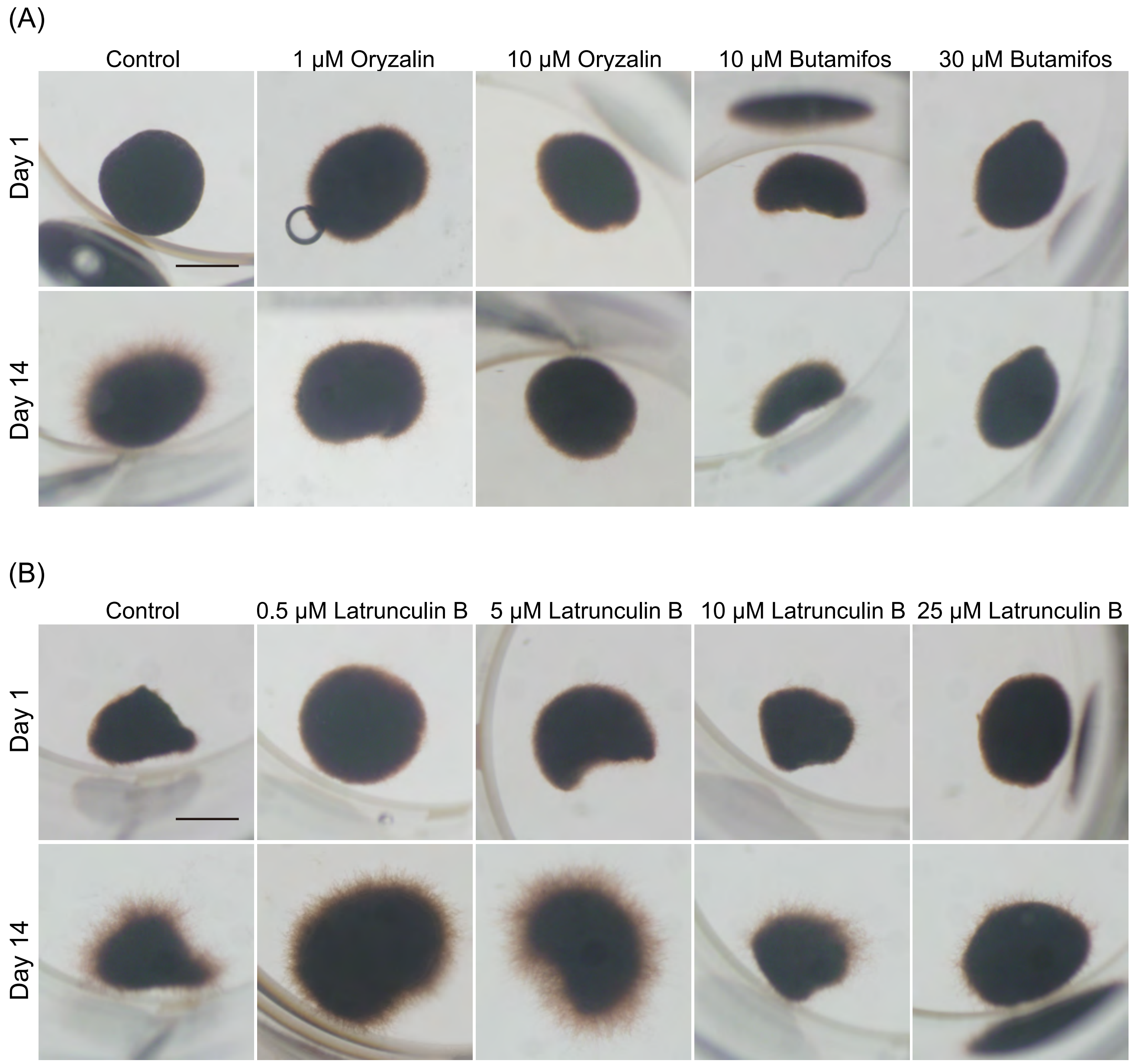

Supplementary Figure 4. Dose-response of conchocelis growth to cytoskeletal drugs. Conchocelis cell clusters were incubated for 14 days with oryzalin and butamifos (A), and Latrunculin B (B). Control indicates the medium containing 1% DMSO. The top and botton panels show the images acquired at 1 and 14 days after inoculation. Scale bars = 2 mm.

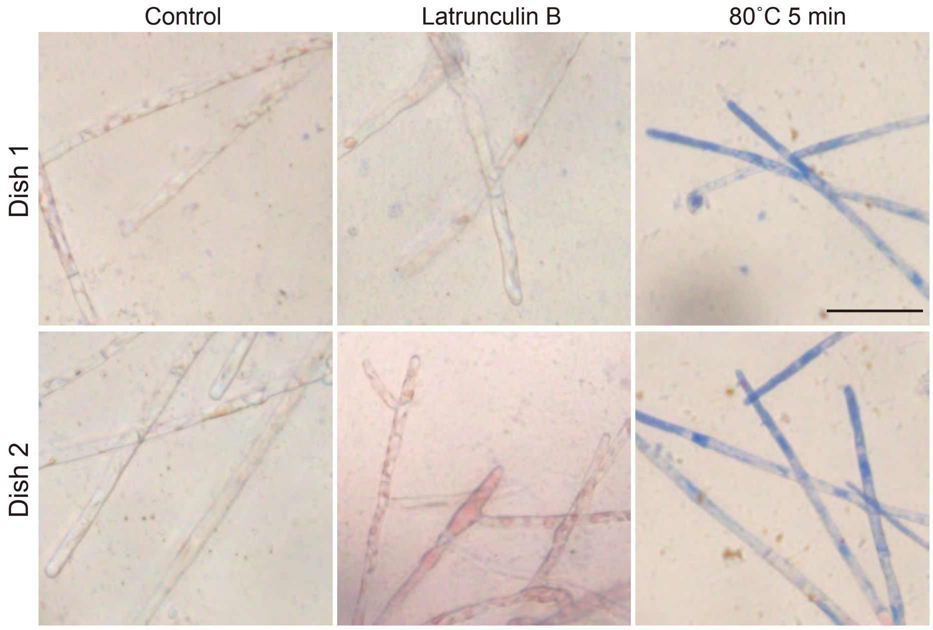

Supplementary Figure 5. The viability of conchocelis cells in the presence of an actin-polymerization inhibitor.

Conchocelis apical cells were incubated with 0.1% DMSO (Control) and 25 μM latrunculin B (Lactrunculin B) for 4 days. Dead cells (80˚C 5 min) were obtained by heating control cells at 80˚C for 5 min prior to staining. These cells were stained with Evans blue. The cells from two different dishes (Dish 1 and Dish 2) are shown. A scale bar = 50 μm.

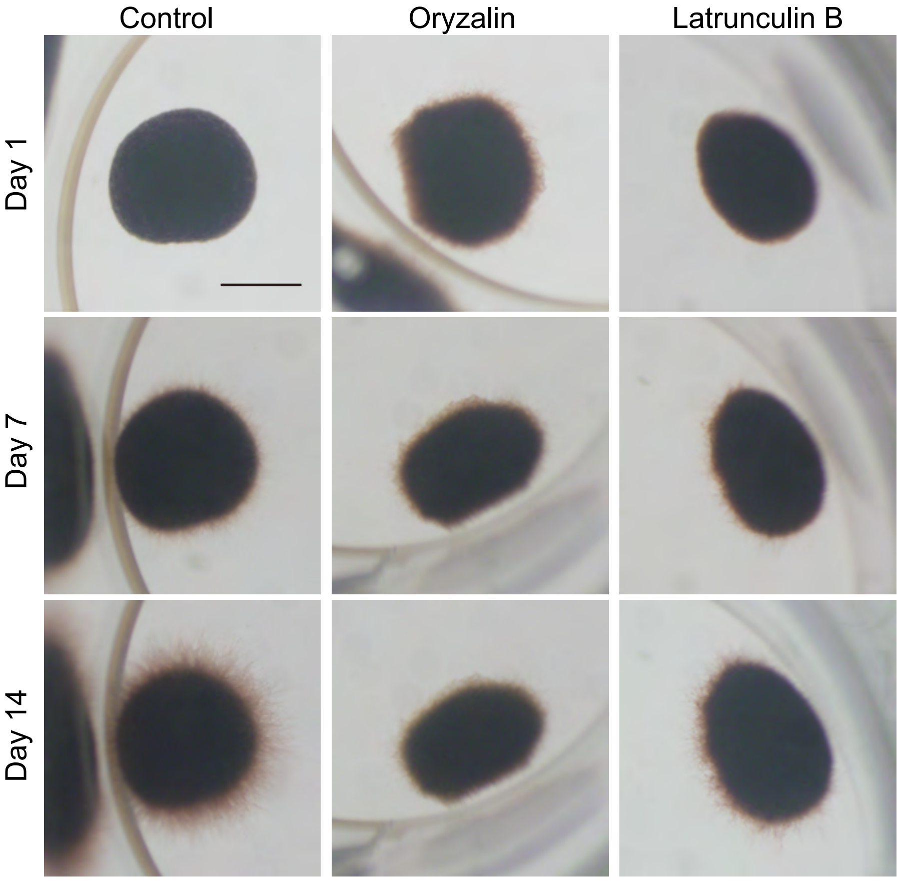

Supplementary Figure 6. Growth of conchocelis cells in the presence of cytoskeletal drugs. Conchocelis cell clusters were incubated for 14 days with 1 μM oryzalin and 25 μM Latrunculin B. Control indicates the medium containing 1% DMSO. The top, middle, and botton panels show the images acquired at 1, 7, and 14 days after inoculation. A scale bar = 2 mm.

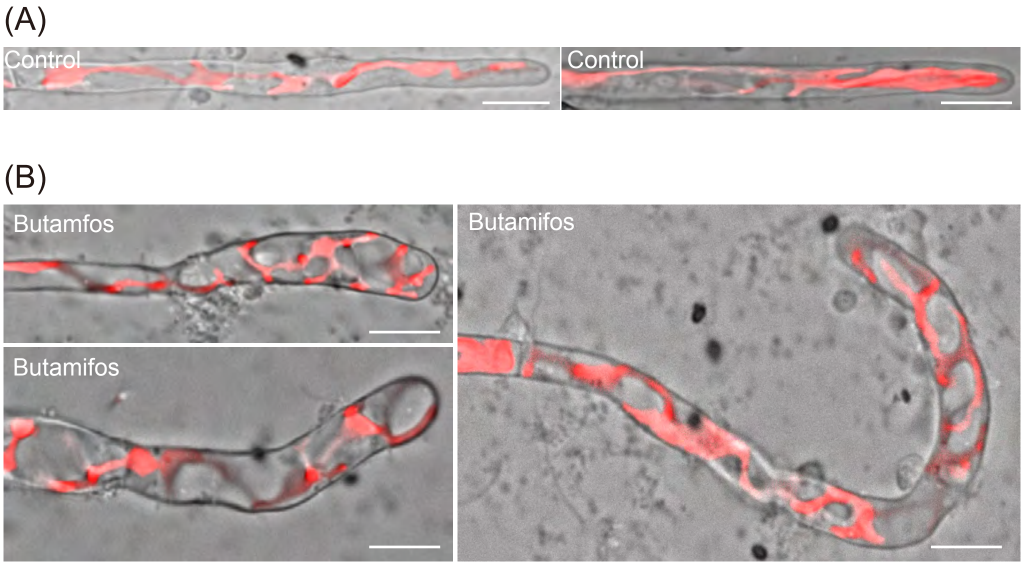

Supplementary Figure 7. Shape of conchocelis cells in the presence of a microtubule -polymerization inhibitor.

Conchocelis apical cells were incubated with 0.1% DMSO as a control (A) and 10 µM butamifos (B) for 4 days. The images are maximum Z-projections of 6 planes (1-µm intervals). The bright field images (gray) and autofluorescence images (red) of chloroplasts are merged. Scale bars = 10 µm.
